# Transanal irrigation: Bridging the gap in treatment for chronic constipation and/or faecal incontinence—A systematic review and management guidance

**DOI:** 10.1111/codi.70274

**Published:** 2025-10-30

**Authors:** Paul F. Vollebregt, Coen I. M. Baeten, Asbjørn M. Drewes, Franco Marinello, Jacopo Martelluci, Mira Mekhael, Karen Nugent, Andreas D. Rink, Harald Rosen, S. Mark Scott, Peter Ernst Slattenschek, Frank Zerbib, Peter Christensen, The ADVISABLE Working Group

**Affiliations:** ^1^ Department of Gastroenterology and Hepatology Amsterdam UMC, Amsterdam Gastroenterology Endocrinology Metabolism Amsterdam the Netherlands; ^2^ Blizard Institute Queen Mary University London London UK; ^3^ Department of Surgery Groene Hart Hospital Gouda the Netherlands; ^4^ Mech‐Sense, Department of Gastroenterology and Hepatology Aalborg University Hospital Aalborg Denmark; ^5^ Colorectal Surgery Unit – Hospital Universitari Vall Hebrón – Universitat Autònoma de Barcelona Barcelona Spain; ^6^ Emergency Sugery/Pelvic Floor Center Careggi University Hospital Florence Italy; ^7^ Collegium Medicum, SAN University Lodz Poland; ^8^ Department of Surgery Aarhus University Hospital Aarhus Denmark; ^9^ Department of Surgery University of Southampton Southampton UK; ^10^ Department of General, Visceral, Vascular and Transplant Surgery Essen University Hospital Essen Germany; ^11^ Sigmund Freud University Centre of Surgery Vienna Austria; ^12^ Department of Gastroenterology, CHU de Bordeaux, Centre Médico‐Chirurgical Magellan, Hôpital Haut‐Lévêque Université de Bordeaux Bordeaux France

**Keywords:** constipation, faecal incontinence, irrigation, TAI

## Abstract

**Aim:**

The aims of the study were to: (1) update a systematic review on the efficacy of transanal irrigation (TAI) in patients with chronic constipation (CC) and/or faecal incontinence (FI); (2) provide guidance on the position of TAI in the treatment pathway of CC and FI; and (3) discuss knowledge gaps and areas of future research.

**Method:**

A collaborative of 11 experts (gastroenterologists, colorectal surgeons, and clinical scientists) from eight European countries was established. The expert group was divided into three main groups, with each group leading a specific section (systematic review, treatment pathway, and knowledge gaps). A previously published systematic review on TAI was updated by conducting an additional search on 12 April 2025. Studies on TAI in specific subgroups (neurogenic bowel dysfunction, low anterior resection syndrome) were excluded.

**Results:**

Sixteen studies involving 1567 (range 16–507) patients were included. Only one underpowered randomised trial was performed, which demonstrated that high‐volume TAI may be more effective than low‐volume TAI in patients with CC. Most other studies (observational) showed improvement in symptoms and/or quality of life to some extent, with a large variation in outcomes used. Treatment discontinuation was reported in 3–57% of patients. The position of TAI in the treatment pathway of CC and FI was proposed, and 13 knowledge gaps were provided.

**Conclusion:**

TAI may be an effective treatment in patients with CC and/or FI. There is a need for randomised controlled trials to study its efficacy and current knowledge gaps.

## INTRODUCTION

Transanal irrigation (TAI) has a long history in the medical field, with the oldest references dating back to the Papyrus Ebers (1500 BC) [1, 2]. The therapy has been used for centuries for a variety of indications, including administration of medications, nutrients, or water to treat many different symptoms including nausea, fever, headache, and colicky pain [1]. Over the past two decades, TAI has emerged as an alternative treatment option in patients with defaecation disorders (chronic constipation [CC] and/or faecal incontinence [FI]) in whom conservative management has failed [3].

TAI is performed by introducing water through the anus to assist in the evacuation of faeces from the colorectum. This results in more complete emptying of the rectosigmoid and left colon in patients with CC [4], and can prevent episodes of FI in‐between irrigations [5].

There is strong evidence supporting the clinical efficacy of TAI in specific patient groups, mainly in those with neurogenic bowel dysfunction (NBD) and low anterior resection syndrome (LARS). A randomised controlled trial comparing TAI with conservative management in patients with spinal cord injury showed significant improvement of symptoms of CC, FI, and quality of life in favour of TAI [6]. In patients who had undergone low anterior resection, four randomised controlled trials have demonstrated that TAI can prevent or improve LARS symptoms and related quality of life [7, 8, 9, 10, 11]. TAI is well acknowledged in previously proposed treatment algorithms of NBD and LARS [12, 13]. However, despite the proven efficacy of TAI in the management of these specific subgroups, the evidence is limited in individuals in the general population who have symptoms of CC and/or FI that are not attributable to NBD/LARS.

A systematic review conducted by Mekhael et al. in 2020 identified 10 studies reporting on the effect of TAI in non‐NBD/non‐LARS patients with CC and/or FI [5]. Most of these studies had a cross‐sectional or retrospective design, had a short follow‐up period, or were conducted in small patient numbers. However, over the past few years, additional studies on the effect of TAI in patients with CC and/or FI have been published. The aims of the current study were to:

1. Update the systematic review by Mekhael et al. [5] with the most recent studies on the efficacy of TAI in non‐NBD/non‐LARS patients with CC and/or FI.
2. Provide management guidance with regard to the position of TAI in the treatment pathway of patients with CC and/or FI in general.
3. Discuss knowledge gaps and areas for future research in this field.

## METHODS

### Advisory board

The ADVIsory BoArd for BoweL disordErs (ADVISABLE) is a collaborative of 11 gastroenterologists, colorectal surgeons, and clinical scientists from several European countries (Austria, Denmark, France, Germany, Italy, the Netherlands, Spain, and the United Kingdom) with an academic and clinical interest in gastrointestinal disorders. A face‐to‐face meeting was held in Copenhagen in April 2024 to set the structure of the project, followed by several online meetings. The expert group was divided into three main groups, with each group leading one of the specific sections of the manuscript (systematic review, treatment pathway, and knowledge gaps).

### TAI equipment and technique

**FIGURE 1:**
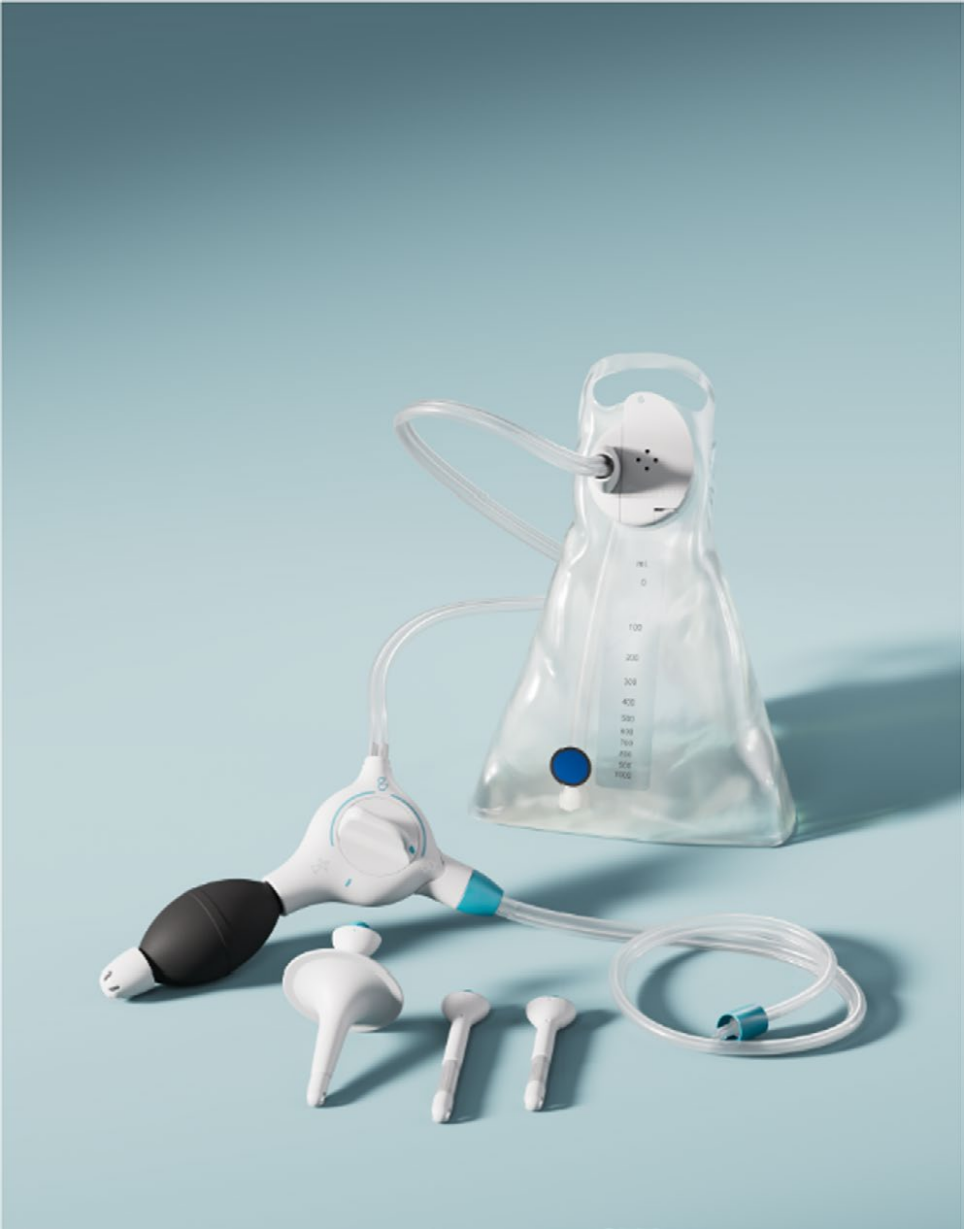
Example of a transanal irrigation device.

A TAI system includes a water bag, a control unit including a (manual/automatic) pump and a rectal catheter (see Figure 1). The position of the rectal catheter is secured by inflation of an integrated rectal balloon or by hand in the case of a cone catheter. Once the catheter is in place, lukewarm tap water is slowly introduced into the colorectum using the pump and the control unit. Once the water is administered, the catheter is removed, which will result in bowel emptying of the inserted water and other bowel contents. It is advised to start TAI with 500–1000 mL daily; frequency and volume can be adjusted until the result is satisfactory.

### Systematic review

For the current systematic review, we updated the results of the systematic review by Mekhael et al. [5] by conducting an additional search on 12 April 2025. This review was conducted according to PRISMA guidelines [14]. We systematically searched the PubMed database for eligible studies. The decision to limit our search to PubMed was because the topic was focused on clinical literature, for which PubMed provides extensive coverage. The terms related to NBD and LARS were removed from the search strategy by Mekhael et al. Aside from this, the search strategy was identical to the one by Mekhael et al. and can be found in the Data S1. One author (PFV) performed the search. The titles and abstracts were screened independently against the inclusion and exclusion criteria by two authors (PFV and FM). Data were extracted from the full‐text articles by one author (PFV), with results checked by another author (PC). The results from the 10 studies identified by the systematic review of Mekhael et al. were adopted with permission from the authors. The review was not registered in a database.

#### Inclusion and exclusion criteria

The systematic review included all studies reporting original data on TAI in patients with CC and/or FI. Studies reporting on TAI in specific subgroups only (e.g. NBD, LARS) were excluded, as well as those studies in which TAI was pooled with other treatment modalities. The study population included adult patients only (≥18 years). All studies had to be published in peer‐reviewed journals, in English or German language.

#### Outcomes

For consistency, the primary and secondary outcomes of the updated systematic review were identical to those used by Mekhael et al. [5] In brief, the primary outcome was defined as the effect of TAI on bowel function measured by patient‐reported outcome measures (PROMs), objective symptom questionnaires, or compliance as a surrogate measure of efficacy. Secondary outcomes included details on TAI (i.e. system type, frequency, volume per irrigation, time per irrigation, trainer type), quality of life measures, discontinuation rate, adverse events, predictive factors for success/failure, and health economics statistics.

#### Risk of bias assessment

The risk of bias was assessed using a modified version of the Downs and Black checklist [15], with all studies being classified as excellent (26–28 points), good (20–25 points), fair (15–19 points), or poor (≤14 points). Quality assessment was independently performed by two authors (PFV and PC). Any disagreements were resolved through discussion between the two authors.

### Treatment pathway and knowledge gaps

The treatment pathway group consisted of four members (CIMB, JM, FM, KN) and the knowledge gap group of four members (AMD, ADR, SMS, FZ). The topics were discussed via online meetings with the entire ADVISABLE working group and further developed by the two specific groups.

## RESULTS

### Systematic review

#### Literature search and study selection

**FIGURE 2:**
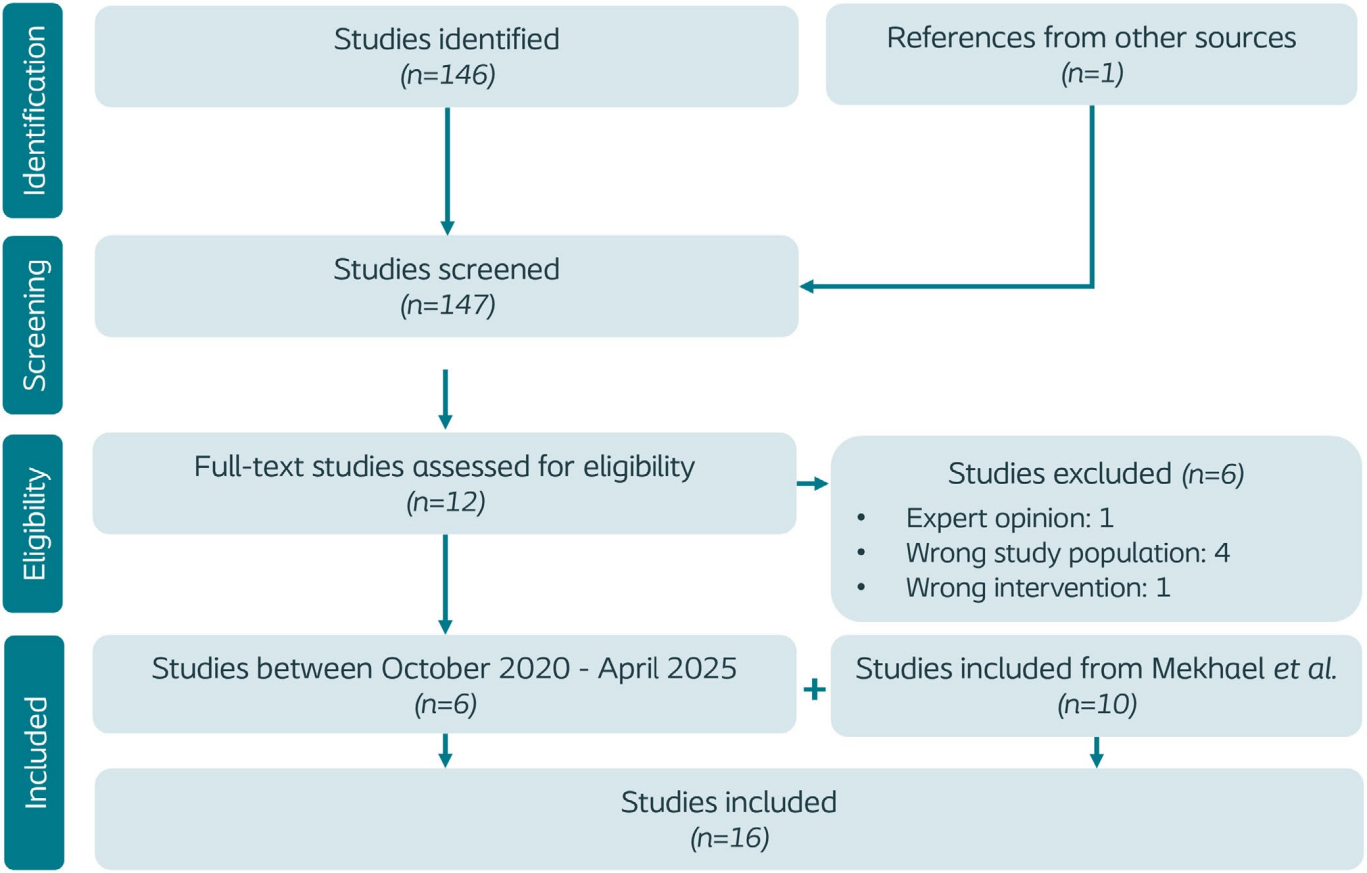
PRISMA flow diagram.

The PRISMA flow diagram in Figure 2 demonstrates the systematic literature search and the process of study selection. In total, 16 studies were identified, of which 6 were published after October 2020 and hence were not reported in the systematic review by Mekhael et al.

#### Study and patient characteristics

The results of the six studies published after October 2020 are shown in Table 1, and the results of the studies reported in the systematic review by Mekhael et al. are shown in Table S1. Overall, studies comprised one randomised trial [16], 10 prospective studies [17, 18, 19, 20, 21, 22, 23, 24, 25, 26, 27], one cross‐sectional study [27] and four retrospective studies [28, 29]. The total number of patients evaluated was 1567, ranging between 16 [17] and 507 [22] patients per study. Eleven studies included patients with CC and / or FI [20, 21, 22, 23, 25, 26, 27, 28, 29, 30, 31], three studies included patients with FI only [17, 18, 24], and two studies included patients with CC only [16, 19]. Follow‐up time ranged between 6 weeks and a mean of 8.5 years [21, 27]. Most studies included both male and female patients [16, 17, 18, 19, 22, 23, 25, 26, 27, 28, 29, 30, 31], one study included females only [24], and sex was not reported in two studies [20, 21]. Median age of the included patients ranged between 42 and 60 years [27, 29].

**TABLE 1:** Included studies between October 2020 and December 2023 on transanal irrigation in patients with chronic constipation and/or faecal incontinence.

Reference	Study design	TAI cohort (total cohort)	Follow‐up time	Inclusion criteria	Patient characteristics	Details on TAI	Bowel function outcome	Quality of life outcome	Discontinuation	Adverse events	Quality assessment
Knowles (2021)	Randomised controlled trial, low‐volume vs. high‐volume TAI	65 randomised, primary outcome available in 43 patients	Primary outcome at 12 weeks, final follow‐up at 52 weeks	CC after failed conservative treatment	Heterogeneous aetiology Age (years), median (IQR): 43 (39–54) Male/female: 7/58	Low‐volume: Qufora IrriSedo Mini High‐volume: various systems including Peristeen (Coloplast) and Qufora IrriSedo Cone 12 weeks: Irrigation volume (mls, IQR) Low‐volume: 144 (70–210) High‐volume: 570 (400–750) Frequency per week (IQR): Low‐volume: 4.0 (1.0–7.0) High‐volume: 3.0 (2.0–4.0) Trained by nurse or physiotherapist	FU 12 weeks Low‐volume: PAC‐SYM (IQR) Baseline: 2.0 (1.4–2.6) FU: 1.8 (1.2–2.1) Global patient improvement score (IQR): 40 (11–70) High‐volume: PAC‐SYM (IQR): Baseline: 2.1 (1.4–2.5) FU: 1.4 (1.0–1.8) Global patient improvement score (IQR): 60 (35–70)	FU 12 weeks Low‐volume: PAC‐QoL (IQR) baseline: 2.4 (1.9–2.9) FU: 2.0 (1.5–2.8). EQ‐VAS (IQR): Baseline: 65 (45–75) FU: 60 (40–75) High‐volume: PAC‐QoL (IQR) baseline: 2.4 (1.8–2.8) FU 1.8 (1.1–2.3) EQ‐VAS (IQR): Baseline: 80 (65–90) FU: 80 (62–90)	FU 52 weeks: 23% discontinued	68 adverse events in 16 patients. Mild: 40 (59%); moderate: 20 (29%). 6 SAEs, none related to treatment	Reporting: 9 External: 0 Internal: 9 Power: 0 Total score: 18
Henderson (2022)	Retrospective	29	Median of 5 months (range 1–12 months)	All patients referred for TAI	Heterogeneous aetiology Age (years), mean (range): 60 (29–87) Male/female: 4/25 *Symptoms*: FI: 7 ODS: 19 STC: 5 FI: 7 Mixed: 3	Qufora IrriSedo mini, cone or klick Irrigation volume (mls) Qufora IrriSedo mini: unknown Qufora IrriSedo cone or klick: 200–1500 Frequency: Between daily and twice weekly Trained by physiotherapist	Telephone interview. St Marks incontinence score Reduction of 35% Cleveland clinic constipation score Reduction of 29%	Not reported	3% discontinued	None	Reporting: 6 External: 0 Internal: 2 Power: 0 Total score: 9
Falletto (2023)	Prospective cohort	289	26 weeks	FC, FI, LARS or a combination	Heterogeneous aetiology Age and sex: not reported Symptoms: FI: 22 CC: 141 LARS: 117 Combination: 9	Peristeen Plus (Coloplast) Irrigation volume (mls), median (IQR): CC: 500 (400–600) FI: 400 (330–500) LARS: 450 (400–650) Frequency per week (IQR): CC: 3.5 (3.0–4.3) FI: 5.0 (3.0–7.0) LARS: 4.0 (3.0–6.0) Trained by healthcare professional	FC: Wexner score (IQR): Baseline: 17 (14–21) FU: 13 (10–17) FI: Wexner score (SD): Baseline: 14 (13–16) FU: 12 (10–14) LARS: LARS score (IQR): Baseline: 37 (34–41) FU: 18 (7–27)	FC: Reduction of PAC‐QoL scores on all domains FI: increase of FI‐QoL scores on all domains LARS: Increase of SF‐36 scores on all domains	8% discontinued: 0% unsatisfactory results, 22% technical difficulties, 30% new symptom, 48% other reasons	Not reported	Reporting: 7 External: 0 Internal: 3 Power: 1 Total score: 11
Vollebregt (2023)	Prospective cohort	114	52 weeks	Rome IV criteria for FC, FI or both, after failed conservative treatment	Heterogeneous aetiology Age (years), median (IQR): 59 (44–66) Male/female: 20/94 Symptoms: FI: 26 CC: 59 Both: 29	Navina Classic or Smart 52 weeks: Irrigation volume (mls), median (IQR): 800 (400–1000) Irrigation time (min), median (IQR): 30 (20–40) Frequency: 12% ≤ 1× per week; 88% 2× per week– 2× per day Trained by: Conservative management nurse	FU 52 weeks: patients with reduction of >30% in symptoms: CC (Cleveland Clinic constipation score): 0% of patients FI (St Marks incontinence score): 42% of patients	FU 52 weeks: CC: reduction of PAC‐QoL scores on all domains. FI: increase of FI‐QoL scores on most domains	55% discontinued: 56% unsatisfactory results, 14% side effects, 6% reasons related to equipment/ Procedure, 27% other reasons	Hospital admission in one patient, no bowel perforation. Side effects at 52 weeks: 20% abdominal pain, 10% anal pain, 10% anal bleeding, 2% autonomic symptoms	Reporting: 8 External: 0 Internal: 4 Power: 1 Total score: 13
Soriano (2023)	Retrospective	40	Mean of 9 months	CC or FI, after failed conservative treatment	Heterogeneous aetiology Age (years), mean (SD): FI: 56 (12); CC: 52 (17) Male/female: 7/33 Symptoms: FI: 20 CC: 19 Both: 1	Peristeen (Coloplast) Irrigation volume (mls), median (range): CC: 800 (400–100) FI: 600 (400–900) Frequency per week (IQR): CC: 1× per 2 days FI: 1× per 2 days Trained using video	CC (Cleveland Clinic constipation score): improvement of 7.6 points FI (Cleveland Clinic incontinence score): improvement of 7.5 points	CC (EQ5D): mean improvement of 32 points FI (EQ5D): mean improvement of 23 points	25% discontinued: 80% unsatisfactory results, 10% expulsion of balloon, 10% pain	13% abdominal pain	Reporting: 9 External: 0 Internal: 2 Power: 0 Total score: 11
Tamvakeras (2023)	Retrospective	18	Median of 28 months (range 5–72 months)	All patients referred for TAI	Heterogeneous aetiology Age (years), median (range): 61 (23–91) Male/female: 3/15 Symptoms: FI: 7 CC:9 Both: 2 LARS: 4 Neurogenic: 3	Peristeen (Coloplast) or Qufora IrriSedo Irrigation volume: not reported Frequency per week: 1x per 2 days Trained at gastrointestinal physiology unit	Patient‐reported bowel symptom bother score (0–10): Baseline: median 9.5 Follow‐up: median 5	Not reported	22% discontinued: 75% unsatisfactory results, 25% improved symptoms	21% anal pain	Reporting: 7 External: 0 Internal: 2 Power: 0 Total score: 9

Abbreviations: CC, chronic constipation; FC, functional constipation; FI, faecal incontinence; FU, follow‐up; IQR, interquartile range; LARS, low anterior resection syndrome; PAC‐QoL, Patient Assessment of Constipation Quality of Life questionnaire; PAC‐SYM, Patient Assessment of Constipation‐Symptoms questionnaire; TAI, transanal irrigation.

#### Primary outcome

In the only randomised trial included in this systematic review, patients with CC were allocated to either low‐volume or high‐volume TAI [16]. The study was underpowered, with the primary outcome (PAC‐QoL score at 3 months) available in 43 patients only (required sample size of 300 patients). At 3 months follow‐up, there was a small reduction of mean PAC‐QoL score from 2.4 to 2.2 points (indicating improvement of quality of life) in the low‐volume group and a larger reduction of 0.5 points in the high‐volume group. Although the difference in favour of high‐volume TAI was small, it was supported by secondary outcomes (including health economics analysis).

To assess clinical efficacy, eight observational studies used a validated bowel‐specific PROM [20, 22, 23, 24, 25, 26, 29, 31], six studies used a non‐validated PROM [17, 18, 19, 21, 27, 30], and one study used compliance as a surrogate measure [28]. Of the eight studies using validated PROMs, seven showed improvement at follow‐up [16, 20, 22, 23, 26, 29, 31]. One study described improvement of FI symptoms in some patients (St Marks incontinence score) but no improvement of CC (Cleveland Clinic constipation score) [25]. However, quality of life (related to both FI and CC) improved in those patients who continued TAI at follow‐up [25]. No improvement of FI symptoms was observed in the last study using a validated PROM [24]. Of those studies using non‐validated PROMs, success was reported in a range between 38 and 73% [17, 18, 19, 21, 27]. Adherence to treatment was used as a measure of success in one retrospective study, in which 43% of patients were still using TAI at 1 year follow‐up [28].

#### Treatment discontinuation

The discontinuation rate was reported in a wide range between the studies (3–57%) [16, 17, 18, 19, 20, 22, 23, 24, 25, 26, 27, 28, 29, 30, 31]. Reasons for discontinuation were most often related to unsatisfactory improvement of symptoms, side effects (e.g. abdominal pain, bloating), reasons related to equipment/procedure (e.g. cumbersome procedure, leakage of water), or patients' personal choice. Important information on whether discontinuation was related to insufficient symptom improvement at the start of the treatment, or loss of response at a later stage was not reported in the included studies.

#### Adverse events

Adverse events were reported in a range between 13% and 59% [16, 19, 22, 23, 25, 27, 28, 30, 31], with the majority being mild. The most frequently reported adverse events were abdominal pain or bloating, anorectal pain, and rectal bleeding. Adverse events were not described in 6 out of 14 studies. Among 1,567 patients evaluated, despite one hospital admission (exact reason unknown) [25], no bowel perforation was reported.

#### Risk of bias assessment

Seven studies were classified as being of good methodological quality [19, 22, 23, 24, 26, 27, 28], two of fair methodological quality [16, 18], and seven of poor methodological quality [17, 20, 21, 25, 29, 30, 31].

### Treatment pathways

**FIGURE 3:**
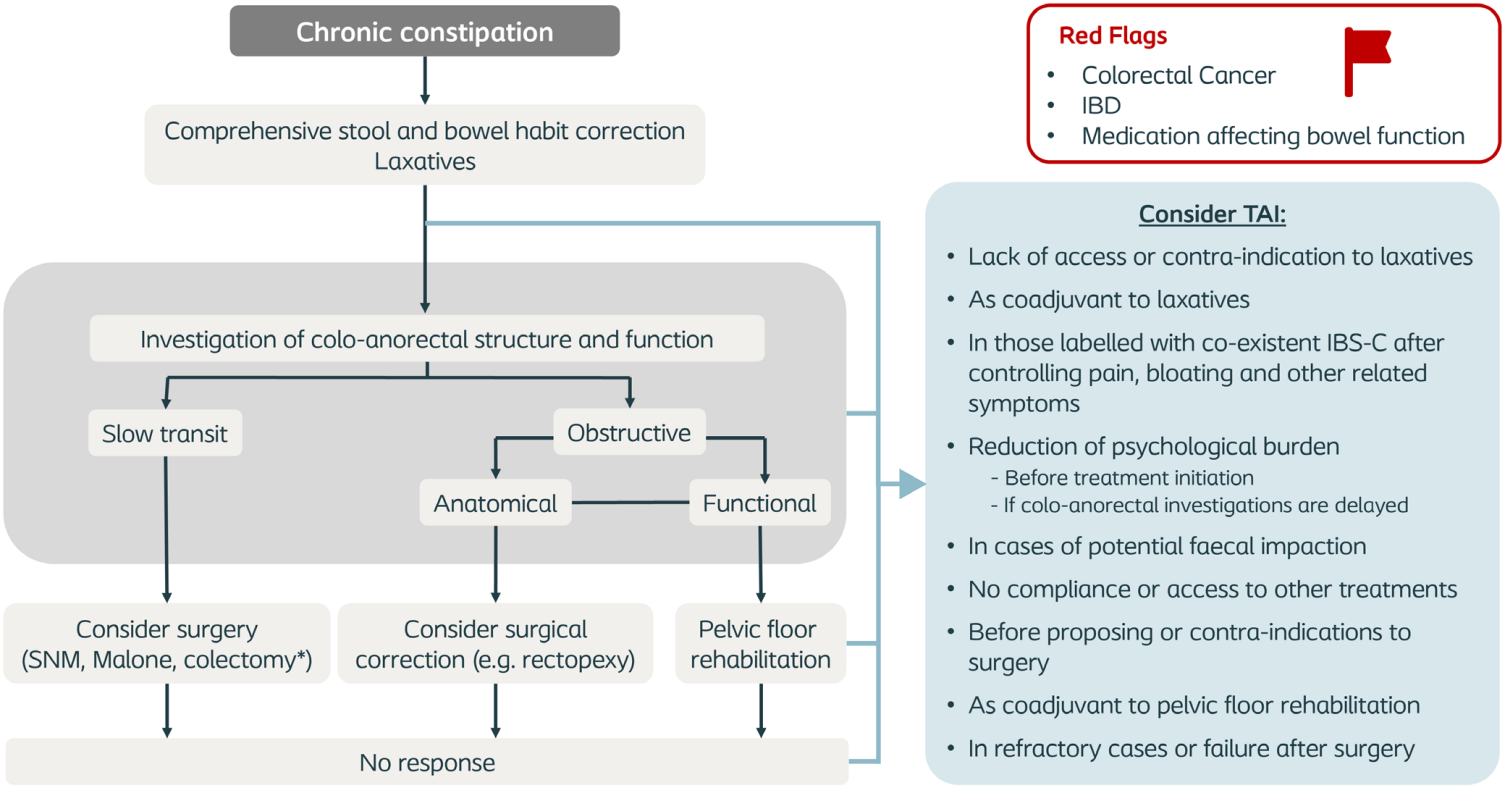
Treatment pathway of patients with chronic constipation. IBD, inflammatory bowel disease; IBS‐C, irritable bowel syndrome with predominant constipation; SNM, sacral neuromodulation; TAI, transanal irrigation. *Colectomy should only be considered after excluding pelvic floor dysfunction, generalised gastrointestinal dysmotility, untreated evacuation disorders, and significant psychological comorbidities, as outlined by Chaichanavichkij et al. [32].

**FIGURE 4:**
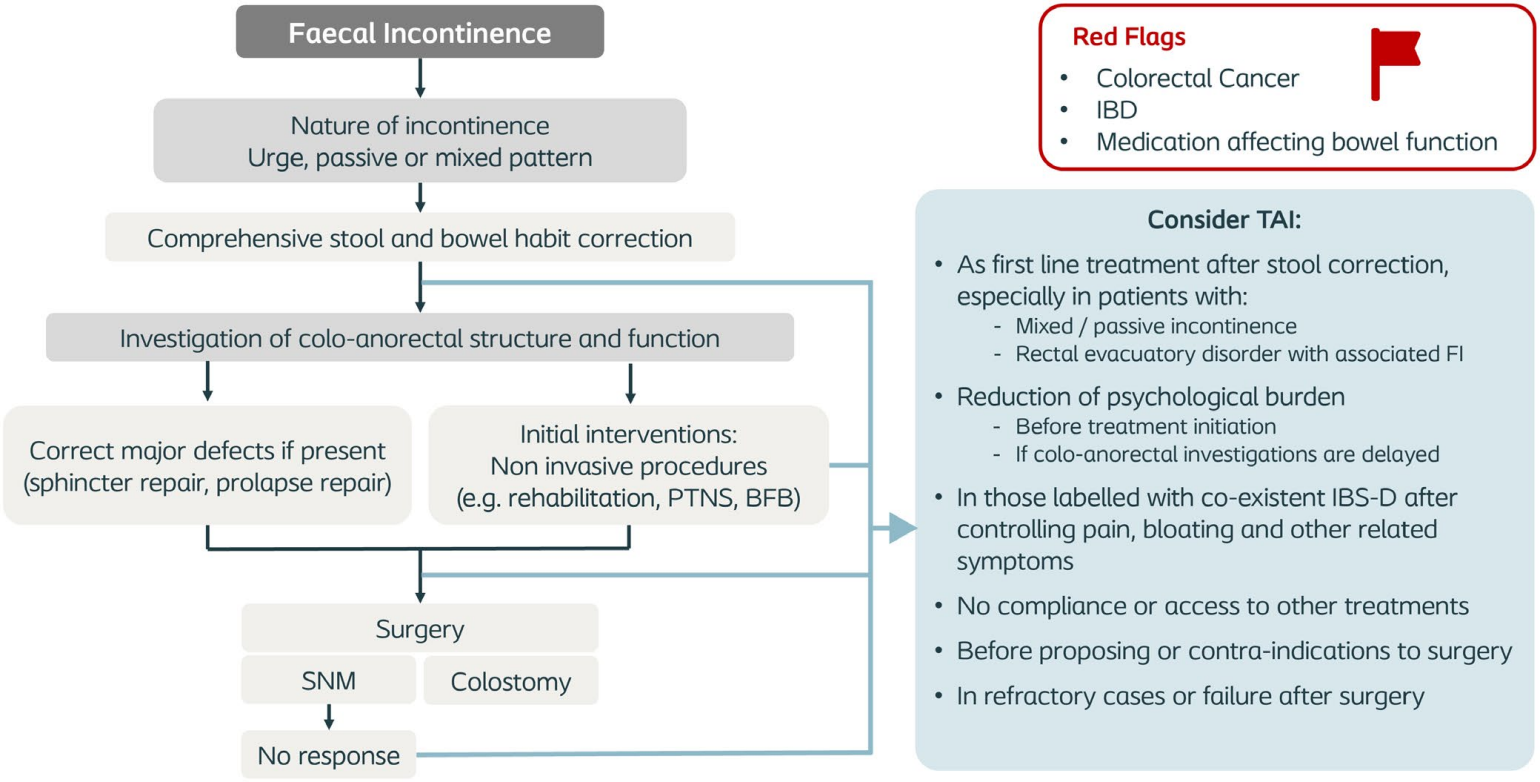
Treatment pathway of patients with faecal incontinence. BFB, biofeedback; FI, faecal incontinence; IBD, inflammatory bowel disease; IBS‐D, irritable bowel syndrome with predominant diarrhoea; PTNS, percutaneous tibial nerve stimulation; SNM, sacral neuromodulation; TAI, transanal irrigation.

Based on the results of the systematic review, and several online discussions and e‐mail correspondences among the authors, the position of TAI in the treatment pathways for the management of CC and FI was identified (Figures 3 and 4, respectively).

## DISCUSSION

This systematic review of 16 studies including 1567 non‐NBD/non‐LARS patients with CC and/or FI demonstrates that TAI may be an effective treatment option in this population. Though most of the studies were observational, they showed an improvement in symptoms and/or quality of life to some extent. Validated PROMs were used in only 56% of the studies. Only one (significantly underpowered) randomised trial was performed, which demonstrated that high‐volume TAI may be more effective than low‐volume TAI in patients with CC. Treatment discontinuation was reported in a wide range between studies (3–57%), which may relate to the equally wide range in follow‐up (6 weeks and a mean of 8.5 years). Adverse events were reported by 13–59% of patients, with the majority being mild. No bowel perforations were reported in the 1567 patients evaluated. There was marked variability in outcomes used between the different studies.

### Treatment pathways

We are aware that the treatment pathways are a pragmatic proposition intended to guide healthcare professionals and lack robust scientific evidence. Accordingly, with the treatment pathways, we do not intend to replace current guidelines on CC and FI.

#### Chronic constipation

CC (Figure 3) is defined by unsatisfactory defaecation, with either infrequent stools, difficulty in passing stool, or both [33]. Professionals need to rule out potential secondary causes, including medication and colorectal cancer, and to evaluate if the patient suffers from predominant slow transit or an evacuation disorder (or both). For this, a radio‐opaque marker study, defaecography, and anorectal manometry (incorporating rectal sensory testing) may be helpful.

In slow‐transit constipation, after initiation of laxative therapy to promote stool formation, TAI can be considered as a coadjuvant to laxatives to facilitate stool discharge and prevent faecal impaction. Moreover, TAI can be useful in patients with contraindications to, or lack of access to laxatives, or in truly refractory cases before proposing surgical procedures, which can include sacral neuromodulation (in those with rectal hyposensitivity [34] or slow‐transit constipation [35]), the Malone procedure, temporary loop‐ileostomy [36] or (in very highly selected cases) colectomy [37]. TAI can also be used in addition to these procedures to treat remaining symptoms.

Patients with evacuation disorders also require correction of stool form and bowel habits. For functional evacuation disorders (i.e. pelvic floor dyssynergia, anismus), pelvic floor rehabilitation should be recommended, and there is growing evidence supporting the use of botox in patients with dyssynergia [38]. For anatomical disorders causing a structural obstruction, such as pelvic organ prolapses (e.g. rectocoele, high‐grade intussusception), potential surgical correction should be assessed. For both subtypes, TAI can be considered after stool form correction or to reduce the burden of symptoms before any further treatments. TAI can also be useful in patients with no access to pelvic floor rehabilitation, those who are not fit for surgery, or if residual symptoms remain following surgery for evacuation disorders.

#### Faecal incontinence

The pathway for FI is shown in Figure 4 (noting that significant symptoms of incontinence and constipation very frequently coexist [39]). After adequate identification of the cause/pathophysiology of FI, management of stool consistency is mandatory. If present, surgical correction of a substantial anal sphincter defect or rectal prolapse should be considered. Patients with FI may benefit from non‐invasive treatments including percutaneous tibial nerve stimulation [40] or biofeedback [41]. In those patients with refractory symptoms, other surgical/endoscopic options should be offered including sacral neuromodulation [42], intrarectal botox injections [43] or a colostomy. TAI can be considered in patients with passive FI secondary to incomplete rectal evacuation, and in those with overflow incontinence. Moreover, TAI may be useful in the reduction of psychological burden before treatment initiation and in cases when anorectal investigations are delayed. TAI can also be considered in patients not fit for surgical procedures or truly refractory or failure cases.

#### Irritable bowel syndrome (IBS)

A large proportion of IBS patients are dissatisfied with their medical therapy [44]. However, TAI has, as yet, not been established as standard therapy due to the complexity of the disease, and it might trigger worsening of symptoms.

### Knowledge gaps

**FIGURE 5:**
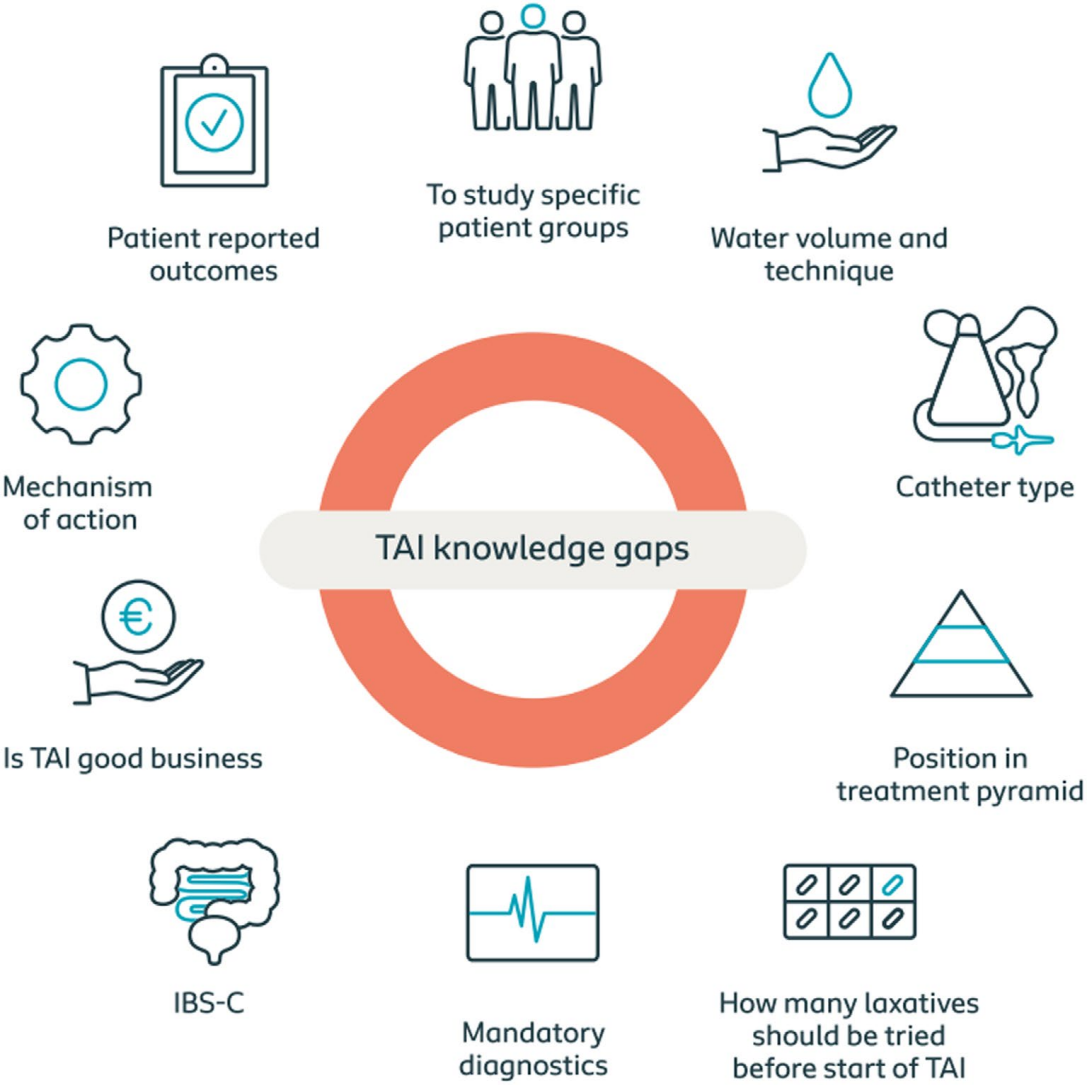
Summary of main knowledge gaps for transanal irrigation.

In Table 2 we provide 13 knowledge gaps and areas for further research in the field of TAI. A summary of the main knowledge gaps is shown in Figure 5. Though the list is not exhaustive and other knowledge gaps can be identified, the authors propose that these are the most unmet needs. A number of different studies should be performed to address these unmet needs. Examples include studies focusing on the underlying mechanisms of action of TAI (e.g. using imaging techniques such as magnetic resonance imaging), careful patient selection (e.g. stratification using colo‐anorectal physiological investigations), the development of TAI‐specific PROMs which require testing for validity and reliability, appropriately designed clinical trials to determine the position of TAI in the treatment pyramid, and health economic studies.

**TABLE 2:** Main knowledge gaps for transanal irrigation.

Questions	Comments
What are the mechanisms of action of TAI?	With regard to the underlying pathophysiology of constipation, in patients with a primary colonic dysmotility manifesting as delayed transit and harder stool consistency, TAI may work through a combination of stool softening and induction of antegrade peristalsis. Conversely, in patients with normal gut motility/transit but incomplete emptying of their (perhaps enlarged) rectum, TAI presumably works through mechanical lavage, induction of defecation and enhancing sensory function, as well as improving patients' symptoms. Pilot trials of TAI using MRI are underway and may shed further light on mechanisms of action
How do we assess the efficacy of TAI?	In many medical trials, QoL scales are often used to assess generic effects, and in TAI, these seem to be more useful in showing efficacy compared with disease/condition‐specific symptom scales (e.g. for constipation). However, better definition of what ‘effective’ TAI is in these patients could be achieved through the development of specific PROMs, which need to be tested for validity and reliability
Which studies are required to better document clinical experience?	It needs to be determined whether broad (e.g. constipation/faecal incontinence/coexistence, or adults vs. children etc.) vs. narrow indications (e.g. very specific patient groups etc.) shall be targeted. Indications can also be interlinked. Accurate phenotyping of included patients is essential and hence sub‐analyses on more specific groups can subsequently be performed according to the underlying pathophysiology. Comparator(s) need to be carefully considered
What volume of water, and which technique should be used?	Optimum technique and volume of irrigant need to be determined. Further, an understanding of whether to use water alone or with added medications (e.g. laxatives) is required (perhaps dependent upon the underlying pathophysiology)
Which catheters should be used in which patients?	Trials (as well as bench‐testing) are needed to determine the optimal catheter type (cone‐shaped vs. balloon etc.). Patients' disabilities must be considered
When in the treatment pyramid shall TAI be used?	TAI may be used: (a) after conventional polypharmacological treatments or immediately after laxatives; (b) as a stand‐alone or complimentary therapy; (c) prior to (or following) nurse‐led conservative therapy or biofeedback; bridge to surgery. Appropriately designed clinical trials are required
Where shall the bar be set for failure of first‐line treatments?	It remains to be determined how many different laxatives a patient needs to try (and which types, maybe in combination) prior to being considered to have failed first‐line treatments and hence be considered a candidate for TAI. Further, early introduction to TAI may prevent symptom progression, but this needs to be tested
Which diagnostics (if any) are mandatory in order to recommend TAI for constipation, faecal incontinence or coexistent symptoms?	Should TAI be recommended on a ‘trial and error’ basis, or should it be recommended more selectively in well‐defined subgroups of patients (based on an understanding of their pathophysiology through diagnostic testing) with a higher likelihood of having a good response? A minimum set of diagnostic investigations needs to be established for basic physiological phenotyping of patients
In patients with constipation (or coexistent constipation/faecal incontinence), which pathophysiological features predict failure of TAI?	It is likely that different biomarkers can be identified to predict treatment responders (and hence non‐responders) using: (a) questionnaires to assess psychological impact; (b) simple clinical measures such as duration of disease; (c) objective findings from colo‐anorectal investigations (e.g. rectal hyposensitivity, pattern of gut transit delay, cause of evacuatory dysfunction etc.). Symptoms of IBS‐C may also impact outcomes. AI models will be needed to handle the many parameters
In patients with faecal incontinence, who are the best candidates for offering TAI?	It remains to be determined whether TAI works best in: (a) those with incontinence in isolation or those with coexistent (?underlying) constipation; (b) those with a hypotensive or normotensive anal sphincter; or (c) those with urge‐related or passive incontinence. Further clinical studies are required to identify more candidate groups
Can TAI be used in patients with IBS‐C?	TAI cannot be recommended before more studies are available as it is not known whether TAI is tolerated. Pain is often the major symptom in IBS‐C patients with constipation and may be a target or perhaps a contraindication for the use of TAI
Where should training best be performed?	It has to be determined whether training is best performed in specialised clinics or GP/district‐nurse‐led clinics. This is likely dependent on the health organisation in the country/region
Is TAI a good business?	There is an unmet need for health economic studies

Abbreviations: AI, artificial intelligence; GP, general practician; IBS‐C, constipation predominant irritable bowel syndrome; MRI, magnetic resonance imaging; PROMs, patient‐reported outcome measures; QOL, quality of life; TAI, transanal irrigation.

## CONCLUSION

In conclusion, this systematic review demonstrates that TAI may be an effective treatment option in patients with CC and/or FI. Nonetheless, there is a need for randomised controlled trials to investigate its efficacy and specific research questions (e.g. comparison of low vs. high‐volume TAI). We propose the position of TAI in the treatment pathway of patients with CC and/or FI and provide knowledge gaps and areas for future research.

## AUTHOR CONTRIBUTIONS

**Paul F. Vollebregt:** Conceptualization; writing – original draft; methodology; visualization; data curation; project administration; formal analysis. **Coen I. M. Baeten:** Conceptualization; writing – review and editing; methodology. **Asbjørn M. Drewes:** Conceptualization; writing – review and editing; methodology. **Franco Marinello:** Conceptualization; writing – review and editing; data curation; methodology. **Jacopo Martelluci:** Conceptualization; writing – review and editing; methodology. **Mira Mekhael:** Writing – review and editing; methodology; data curation. **Karen Nugent:** Conceptualization; writing – review and editing; methodology. **Andreas D. Rink:** Conceptualization; writing – review and editing; methodology. **Harald Rosen:** Conceptualization; writing – review and editing; methodology. **S. Mark Scott:** Conceptualization; writing – review and editing; methodology. **Peter Ernst Slattenschek:** Conceptualization; writing – review and editing; data curation. **Frank Zerbib:** Conceptualization; writing – review and editing; methodology. **Peter Christensen:** Conceptualization; methodology; writing – review and editing; data curation; supervision.

## FUNDING INFORMATION

Paul F. Vollebregt received financial support from Coloplast A/S to write the manuscript.

## CONFLICT OF INTEREST STATEMENT

Paul F. Vollebregt received honoraria from Coloplast A/S and Qufora as an Advisory Board member. Coen I. M. Baeten received honoraria from Coloplast A/S for consultancy, teaching, and as an Advisory Board member. Asbjørn M. Drewes received an unrestricted research grant from Shionogi and consultancy honoraria from Coloplast A/S, Pharmanovia, Pangenic, and Shionogi. Franco Marinello received honoraria for consultancy, Advisory Board member, and speaker from Coloplast A/S and Medtronic. Jacopo Martelluci received honoraria for consultancy, Advisory Board member, and speaker from Coloplast A/S, Medtronic, and Servier. Andreas D. Rink received honoraria for consultancy, Advisory Board member, and speaker from Coloplast A/S, Applied Medical, Dr. Falk Pharma, and Sanofi. Harald Rosen received honoraria from Coloplast A/S for consultancy, teaching, and as an Advisory Board member. S. Mark Scott has received honoraria from Laborie for teaching and from Coloplast A/S for consultancy and as an Advisory Board member/speaker. Frank Zerbib received honoraria for consultancy, Advisory Board member, and speaker from Coloplast A/S, Dr. Falk Pharma, Sanofi, BMS, Bioprojet, AstraZeneca, Medtronic, and Reckitt. Peter Christensen received honoraria from Coloplast A/S for consultancy, teaching, and as an Advisory Board member, and also from Wellspect and Qufora.

## ETHICS STATEMENT

Ethical approval was not required.

## Supporting information


Data S1:

## Data Availability

Data sharing is not applicable to this article as no new data were created or analysed in this study.
