# Supplementary material for: Transanal irrigation: Bridging the gap in treatment for chronic constipation and/or faecal incontinence—A systematic review and management guidance
Source: Colorectal Dis. 2025 Oct 30;27(11):e70274. doi: 10.1111/codi.70274 (PMC12575420; doi:10.1111/codi.70274)
Supplement: Supplementary file 1 — Data S1: [file CODI-27-0-s001.docx]

**SUPPLEMENTARY DOCUMENT**

**Search strategy**

(bowel[Text Word] OR fecal[Text Word] OR faecal[Text Word] OR incontinence[Text Word] OR constipation[Text Word] OR intestinal[Text Word] OR gastrointestinal[Text Word] OR stool[Text Word] OR "Feces"[Mesh] OR "Neurogenic Bowel"[Mesh] OR "Intestines"[Mesh] OR "Fecal Incontinence"[Mesh] OR "Defecation"[Mesh] OR "Constipation"[Mesh] OR "Lower Gastrointestinal Tract"[Mesh] OR "Gastrointestinal Diseases"[Mesh] OR "Gastrointestinal Tract"[Mesh] OR "Intestine, Large"[Mesh] OR "Gastroenterology"[Mesh] OR "Flatulence"[Mesh] OR "Digestive System Diseases"[Mesh] OR "Digestive System"[Mesh] AND "Constipation"[Mesh] OR "Constipation"[Text Word] AND (english[Filter])))

OR (bowel[Text Word] OR fecal[Text Word] OR faecal[Text Word] OR incontinence[Text Word] OR constipation[Text Word] OR intestinal[Text Word] OR gastrointestinal[Text Word] OR stool[Text Word] OR "Feces"[Mesh] OR "Neurogenic Bowel"[Mesh] OR "Intestines"[Mesh] OR "Fecal Incontinence"[Mesh] OR "Defecation"[Mesh] OR "Constipation"[Mesh] OR "Lower Gastrointestinal Tract"[Mesh] OR "Gastrointestinal Diseases"[Mesh] OR "Gastrointestinal Tract"[Mesh] OR "Intestine, Large"[Mesh] OR "Gastroenterology"[Mesh] OR "Flatulence"[Mesh] OR "Digestive System Diseases"[Mesh] OR "Digestive System"[Mesh] AND "Fecal Incontinence"[Mesh] OR incontinence[Text Word]

AND (english[Filter])) AND (english[Filter])) AND (irrigation OR "Therapeutic Irrigation"[Mesh] AND (english[Filter])) AND (english[Filter]) AND (english[Filter])

| **Supplementary Table 1.** Results from the 10 studies identified by the systematic review of Mekhael *et al.^1^* | | | | | | | | | | | |
| --- | --- | --- | --- | --- | --- | --- | --- | --- | --- | --- | --- |
| **Reference** | **Study design** | **TAI cohort (total cohort)** | **Follow-up time** | **Inclusion criteria** | **Patient characteristics** | **Details on TAI** | **Bowel function outcome** | **Quality of life outcome** | **Discontinuation** | **Adverse events** | **Quality assessment** |
| Briel 1996 | Prospective cohort | 16 | Median of 18 months | Impaired continence | Heterogeneous aetiology Age (years), median (range): 52 (25–72) Male/female: 5/11 FI: 16 | System unspecified Irrigation time (min), median (range): 30 (10–90) Irrigation frequency: 87% ≥ 1 time a day Trained by enterostomal therapist | 38% reported a successful outcome | Not reported | 6 (38%) patients discontinued | Not reported | Reporting: 4 External: 1 Internal: 4 Power: 0 Total score: 9 |
| Crawshaw 2004 | Prospective cohort | 48 | Median (range): 11 (4–27) months | Absence of correctable pathology or the failure of medical and surgical treatment | Heterogeneous aetiology Age (years), median (IQR): 54 (41–61) Male/female: 13/35 Symptoms: FI: 33 CC: 15 | Equipment adapted from a Coloplast Stoma Irrigation set (Coloplast A/S, Denmark) Irrigation volume: 1500 mL Irrigation frequency: 5% twice a day, 38% daily, 17% on alternate days, 15% every 3–7 days, 19% as required Trained by specialist nurse | Bowel control, visual analogue scale: Successful response to TAI in 24 (50%) patients. Bowel rating among these 24 patients, VAS 100 maximum (100 = full control), median (IQR): Pre: 15 (3–24) Post: 50 (34–65) | QoL among 24 patients with successful outcome, median (IQR): 59.16 (46.55–67.43) No difference compared to the 24 patients without successful response | 4 (8%) patients discontinued: 50% unacceptable, 50% relief of symptoms with rectopexy | Not reported | Reporting: 8 External: 2 Internal: 8 Power: 0 Total score: 18 |
| Gardiner 2004 | Prospective cohort | 57 | 6 weeks |  | Symptoms: FI: 16 CC: 41 | Not reported | Proportion of patients with successful outcome: FI: 75% CC: 51% Slow transit CC (n = 15): 57% Obstructed defaecation (n = 26): 42% | Not reported | FI: 2 (12.5%) patients discontinued: 6.25% not severe enough symptoms to continue TAI, 6.25% still under review | Not reported | Reporting: 2 External: 1 Internal: 4 Power: 0 Total score: 7 |
| Cazemier 2007 | Cross-sectional | 40 | Time (y) using irrigation, mean (range): 8.5 (2.5–18) | FI or CC TAI No response to medical treatment or biofeedback | Heterogeneous aetiology  Includes NBD FI: 28 Age (years): 42 Male/Female: 5/23 CC: 12 Age (years): 45 Male/Female: 3/9 | Iryflex® (B. Braun Medical A/S, Germany) Irrigation volume: 500–1000 mL Frequency: 32% daily, 36% 3 times/week, 32% twice or less/week | 25 (63%) patients still used TAI Overall satisfaction (n = 40): 29 (73%) Actual users (n = 25), satisfaction: 22 (88%) | Not reported | Overall, 15 (38%) discontinued: FI: 5 (29%) CC: 7 (58%) | Side effects: 37.5% abdominal cramps | Reporting: 9 External: 3 Internal: 10 Power: 0 Total score: 22 |
| Koch 2008 | Prospective cohort | 39 | 3, 6 and 12 months | FI or CC or both after failed conservative treatment or after (partially) unsuccessful surgical treatment for defaecation disorder | Heterogeneous aetiology Age (years), mean (SD): 58 (13.5) Male/Female: 13/26 Symptoms: FI: 18 CC: 11 FI + CC: 10 | Biotrol® Irrimatic pump (B. Braun Medical A/S, Germany) or irrigation bag Braun (B. Braun Medical A/S, Germany) 1-year FU: Irrigation volume (L), mean (SD): 1.75 (0.79) Irrigation time (min), mean (SD): 36.39 (16.02) Frequency (time/day), mean (SD): 1.1 (0.49) Trained by physician | 3 months FU, number (%) pseudo continent: FI: 11 (61%) (p < 0.001) FI + CC: 6 (60%) (p = 0.009) Baseline compared with 1-year FU: FI: Park’s score [61]: 3.61 (0.5) to 1.6 (0.92) (p < 0.005) CCCS: Feeling of incomplete evacuation: 1.60 (2.47) to 2.75 (1.36) (p = 0.036) | Improvement in overall QoL measured with SF-36 and the FIQLS (p = 0.012) | 9 (23%) patients discontinued: 78% unsatisfactory results, 22% appendicostomy | 23 (59%) experienced side effects: 7% leakage after irrigation, 16% abdominal cramps, 22% abdominal bloating, 13% combination of the above side effects, 2% other | Reporting: 11 External: 2 Internal: 8 Power: 0 Total score: 21 |
| van der Hagen 2012 | Multicentre non-randomised trial | 35 (70) | 6 months | History of birth trauma Passive faecal incontinence CCIS ≤ 8 after anal sphincter exercise and biofeedback Defect of the internal anal sphincter | Sphincter damage after birth trauma Age (years), mean (range): 53 (38–74) | REPROP® Clyster Trained by specialist nurse | In 3 (9%) patients faecal incontinence resolved completely Baseline 6-month FU: CCIS, average number of days per week with incontinence for solid or liquid stools, and average number of pads used did not change significantly | Not reported | 3 (9%) patients discontinued | No severe adverse effects | Reporting: 11 External: 2 Internal: 7 Power: 0 Total score: 20 |
| Vollebregt 2016 | Prospective cohort | 60 | Median FU: 12 months | Chronic defaecatory disorders not responding to conservative treatment | Heterogeneous aetiology Includes NBD and colorectal surgery Age (years), median (range): 49 (21–74) Male/female: 15/45 Symptoms: FI: 8 CC: 44 FI + CC: 8 | Peristeen® (Coloplast A/S, Denmark) or Biotrol® Irrimatic pump (B. Braun Medical A/S, Germany) Irrigation volume (mL), median (range): 875 (250–2200) Frequency: 6% twice/day, 52% daily, 33% every second day, 6% when needed Trained by enterostomal therapist | First FU: FIQLS score did not differ between patients continuing or discontinuing TAI | First FU: Using SF-36 patients continuing TAI had more energy and were less fatigued compared with patients discontinuing TAI (p = 0.01) Patients continuing TAI had a tendency to have a higher SF-36 social functioning and a higher total SF-36 score, but this was non-significant | 33 (55%) of patients had discontinued at the first FU, 37 (62%) at second FU and 38 (63%) at last FU | Not reported | Reporting: 10 External: 3 Internal: 8 Power: 0 Total score: 21 |
| Juul 2017 | Prospective cohort | 507 | Mean (range): 1.06 (0.52–1.46) years | Intractable FI and/or CC with unsatisfactory results after conservative treatment | Heterogeneous aetiology Includes NBD and anorectal surgery Age (years), median (range): 56 (19–86) Male/female: 84/423 Symptoms: FI: 238 CC: 171 FI + CC: 98 | Coloplast irrigation bag® /Colotip® (Coloplast A/S, Denmark) (majority), Coloplast irrigation bag® (Coloplast A/S, Denmark)/Qufora cone® (MBH International A/S), Aqua colon enema tip with silicone balloon ch 24® (Runfold Plastics Ltd., UK) or Peristeen® (Coloplast A/S, Denmark) Irrigation volume (mL), median (IQR): 1000 (750–1000) Irrigation time (min), median (IQR): 20 (15–30) Frequency: 35% daily, 16% every second day, 20% 2–3 times/week, 21% < once a week Self-administered 99%, assistance 1% Trained by specialist nurse | Patients with FI, pre-/post-treatment, mean change (95% CI): 11-point Likert, FI: 2.7 (2.2–3.2) (p < 0.001) CCIS: 2.2 (1.6–2.8) (p < 0.001) FIGS score: 2.2 (1.5–2.9) (p < 0.001) 65% improvement of FI, 29% stability, and 6% deterioration. Patients with CC, pre/posttreatment, mean change (95% CI): 11-point Likert, CC: 1.6 (0.9–2.4) (p < 0.001) CCCS: 1.9 (1.1–2.7) (p < 0.001) ODS score: 3.3 (2.0–4.5) (p < 0.001). 48% improvement of CC, 40% stability and 12% deterioration. | Patients with FI and CC, pre- /post-treatment, mean change (95% CI): 11-point Likert, QoL: 1.8 (1.4–2.2) (p < 0.001) | 174 (34%) discontinued: 49% inefficacy, 18% dislike treatment, 16% symptoms resolved, 13% time consumption, 12% side effects, 8% practical problems, 21% other, 8% undetermined | 120 (58%) patients experienced side effects: 23% abdominal pain, 15% anorectal pain, 6% chills/ shivering, 11% nausea, 8% dizziness, 13% sweating | Reporting: 11 External: 2 Internal: 8 Power: 0 Total score: 21 |
| Bildstein 2017 | Retrospective | 108 | 1-year FU | FI or CC Refractory to conservative treatment | Heterogeneous aetiology Includes NBD Age (years), mean (range): 55 (18–83) Male/female: 21/87 Symptoms CC: 51 FI + CC: 47 FI: 10 | Peristeen® (Coloplast A/S, Denmark) Trained by specialist nurse | 1-year FU: 46 (42.6%) patients still irrigated 62 (57%) discontinued: 44 had discontinued, 5 failed during first training, 12 lost to follow-up and 1 died | Not reported | Reasons for discontinuation: 36.4% technical problems, 40.9% inefficacy, and 22.7% constraints (primary time consuming) Median (range) time before discontinuation: 3 (0.2–11) months | 25 (54.3%) reported minor 47 minor and self-limiting adverse events: 34% leakage of fluid around catheter, 29.9% pain when inserting catheter or water, 19.1% catheter expulsion, 10.6% rectal balloon burst, 6.4% water retention | Reporting: 11 External: 3 Internal: 9 Power: 0 Total score: 23 |
| Etherson 2017 | Prospective cohort | 102 | Length of therapy use, median (range): 30.15 (1–460) weeks | Fulfilled Rome II criteria Past or present TAI treatment Received TAI for chronic idiopathic constipation (CIC) Failed all medical and behavioural therapies | Chronic idiopathic constipation (CIC) Age (years), median (range): 45 (25–84) Male/female: 7/95 Duration (years) of CIC, mean (SD): 21.8 (16.9) | Peristeen® (Coloplast A/S, Denmark) (majority), Qufora® (MBH International A/S) Biotrol® Irrimatic pump (B. Braun Medical A/S, Germany) Frequency: on average every second day | Overall symptom improvement: Bowel frequency: 42% Clearance of rectum: 63% Abdominal pain: 48% Bloating: 49% General well-being: 65% Awareness of urge: 25% Overall satisfaction with TAI was reported by 67% as either moderately better or very much better | Not reported | 48 (47%) patients discontinued | 22 (22%) patients experienced side effects: 6% rectal bleeding, 3% painful irrigations, 2% painful haemorrhoids, 2% new anal fissure, 10% bursting balloons, 3% splitting of catheter | Reporting: 10 External: 2 Internal: 8 Power: 0 Total score: 20 |
